# Prediction of essential binding domains for the endocannabinoid *N*-arachidonoylethanolamine (AEA) in the brain cannabinoid CB1 receptor

**DOI:** 10.1371/journal.pone.0229879

**Published:** 2021-06-28

**Authors:** Joong-Youn Shim

**Affiliations:** Department of Physical Sciences, School of Arts and Sciences, Dalton State College, Dalton, Georgia, United States of America; UMR-S1134, INSERM, Université Paris Diderot, INTS, FRANCE

## Abstract

Δ^9^-tetrahydrocannabinol (Δ^9^-THC), the main active ingredient of *Cannabis sativa* (marijuana), interacts with the human brain cannabinoid (CB1) receptor and mimics pharmacological effects of endocannabinoids (eCBs) like *N*-arachidonylethanolamide (AEA). Due to its flexible nature of AEA structure with more than 15 rotatable bonds, establishing its binding mode to the CB1 receptor is elusive. The aim of the present study was to explore possible binding conformations of AEA within the binding pocket of the CB1 receptor confirmed in the recently available X-ray crystal structures of the CB1 receptor and predict essential AEA binding domains. We performed long time molecular dynamics (MD) simulations of plausible AEA docking poses until its receptor binding interactions became optimally established. Our simulation results revealed that AEA favors to bind to the hydrophobic channel (HC) of the CB1 receptor, suggesting that HC holds essential significance in AEA binding to the CB1 receptor. Our results also suggest that the Helix 2 (H2)/H3 region of the CB1 receptor is an AEA binding subsite privileged over the H7 region.

## Introduction

Δ^9^-tetrahydrocannabinol (Δ^9^-THC), the main active ingredient of *Cannabis sativa* (marijuana), interacts with the brain cannabinoid (CB1) receptor and elicits a wide range of neurological, psychological and biological effects [1]. Continuous marijuana use may increase risks of addiction, chronic pain, mood disorders and birth defects [2, 3].

Recently determined X-ray crystal structures of the CB1 receptor in complex with various ligands [4–7] have revealed the detailed receptor interactions with the bound ligand. Toward understanding molecular mechanisms of marijuana action, these X-ray crystal structures have also shed light on how the ligand activates the receptor upon binding at the molecular level. It is seen in the X-ray crystal structure of the classical cannabinoid full agonist AM11542-bound CB1 receptor [6] that the dimethyl heptyl (DMH) tail of the ligand binds the hydrophobic channel (HC), disrupting the toggle switch of Phe200/Trp356 a pair of key aromatic residues that has been proposed to be required for CB1 receptor activation [8]. HC appears to be conserved not only in the CB1 receptor but also in other related lipid receptors [9, 10]. The classical cannabinoid Δ^9^-THC is expected to bind the CB1 receptor in a way similar to AM11542. Thus, it is likely that the known partial agonistic activity of Δ^9^-THC [11, 12] is attributed to its pentyl tail moiety that binds HC but not as tightly as the DMH tail of AM11542.

**Fig 1:**
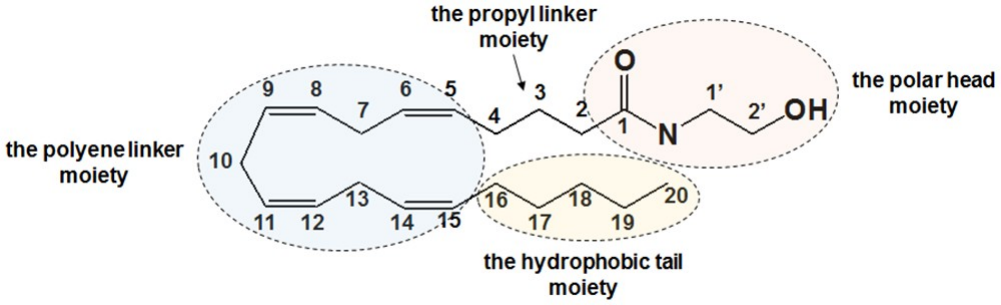
Structure of anandamide (AEA). The structure of AEA consists of three moieties, including the polar head moiety, the polyene linker moiety and the hydrophobic tail moiety.

**Fig 2:**
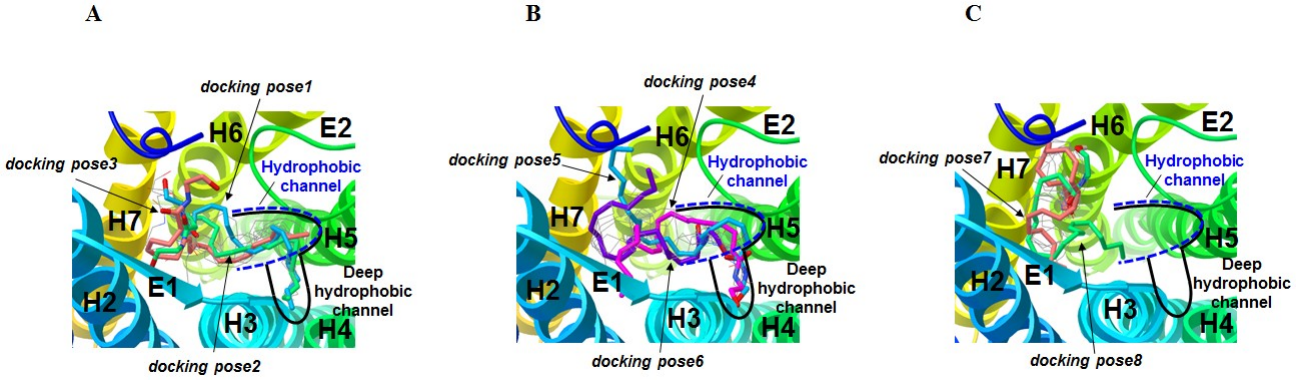
Selection of eight representative AEA docking poses from AutoDock docking poses. (A) AEA docking pose Group **1**: *docking pose1* (in cyan), *docking pose2* (in green) and *docking pose3* (in orange). (B) AEA docking pose Group **2**: *docking pose4* (in magenta), *docking pose5* (in cyan) and *docking pose6* (in purple). (C) AEA docking pose Group **3**: *docking pose7* (in orange) and *docking pose8* (in green).

Just like Δ^9^-THC, endogenous lipid ligands such as *N*-arachidonylethanolamide (AEA) (Fig 1) and 2-arachidonoylglycerol (2-AG), known as endocannabinoids (eCBs), also interact with the CB1 receptor [1]. AEA was isolated from porcine brain [13] and 2-AG from canine intestines [11]. These eCBs are known to be produced only when biologically demanded [14, 15]. While 2-AG is known to be a full agonist at CB1 receptors [16], AEA is a partial agonist at CB1 receptors [17] just like Δ^9^-THC but somewhat more potent than Δ^9^-THC in activating the CB1 receptor [1]. A common structural feature of eCBs is a long lipid chain containing the polyene linker moiety and the pentyl tail moiety (Fig 1), which makes eCBs extremely flexible and allows them to adopt millions of conformations. Identification of the bioactive conformation of AEA at the CB1 receptor can be quite elusive due to its potential to adopt many low-energy binding conformations only a few of which would be responsible for receptor activation. Without any known X-ray crystal structure of the AEA-bound CB1 receptor, the nature of binding interactions of AEA with the CB1 receptor remains poorly understood.

Our initial motivation of the present study was due to some intriguing results from recent studies demonstrating that the CB1 allosteric modulators (AMs) such as lipoxin A_4_ and ZCZ011 selectively enhance the AEA-activated CB1 receptors [18–20]. As the first step toward understanding how the CB1 AMs allosterically enhance AEA-activated CB1 receptors, we felt imperative to determine the binding conformation of AEA responsible for CB1 receptor activation, particularly how AEA interacts with the conserved HC. In the present study, by using a combination of molecular docking and molecular dynamics (MD) simulation approaches, we explored many possible binding conformations of AEA within the binding pocket of the CB1 receptor and identified essential AEA binding domains. Our results indicate that HC interactions are crucial for AEA binding to the CB1 receptor. Our results also suggest that the Helix 2 (H2)/H3 region of the CB1 receptor is an AEA binding subsite privileged over the H7 region.

## Methods

### Determination of the AEA binding models

A low-energy ligand structure of AEA was obtained by performing the conformational analysis by using the MMFF molecular mechanics force field [21] implemented in the SPARTAN computational modeling package (Spartan’18, Wavefunction, Inc. Irvine, CA). Initial docking poses of AEA were generated by using AutoDock4 [22]. For the receptor template, the CB1 receptor in the HU210-bound CB1-Gi complex model [23] refined according to the X-ray crystal structure of the AM11542-bound CB1 receptor [6] was used. The validity of the CB1 receptor model was partly confirmed by the overlay of the classical cannabinoid HU210 bound to the CB1 receptor in the refined CB1-Gi complex model to AM11542 in the X-ray crystal structure of the AM11542-bound CB1 receptor [6], which shows almost identical positions (see S1 Fig). For exploring AEA binding to the CB1 receptor, a grid box was created by setting 60 grid points in the x and y dimensions and 56 grid points in the z dimension with 0.375 Å spacing between grid points (i.e., a box of 22.5 Å x 22.5 Å x 21.0 Å) such that it covered the entire orthosteric binding pocket region. The position of the center of the grid box was guided by AM11542 bound to the CB1 receptor in the X-ray crystal structure of the AM11542-bound CB1 receptor [6]. A typical setting of docking parameters for performing AutoDock runs using a hybrid global-local Lamarkian genetic algorithm (LGA) [24] included: the rate of gene mutation (0.02), rate of crossover (0.8), GA window size (10), the number of individuals in population (150), the maximum number of energy evaluations in each run (25,000,000), the maximum number of generations (27,000) and the number of LGA docking runs (10). Only the ligand was allowed to freely move inside the grid box while the protein was rigidly fixed in position. The resulting docking poses were evaluated by the AutoDock4 scoring function [25]. AutoDock runs were performed more than one hundred times using the best scoring docking pose from the previous run as the starting pose for the next run. For every run the same grid box was used.

The best scoring docking poses obtained from the above AutoDock runs were overlaid to AM11542 bound to the CB1 receptor in the X-ray crystal structure [6]. Then, depending upon how AEA interacted with HC, where the DMH tail of AM11542 was occupied in the X-ray crystal structure of the AM11542-bound CB1 receptor [6], they were clustered into three distinct AEA docking pose groups: 1) AEA docking pose Group **1** where the hydrophobic tail moiety of AEA occupied HC; 2) AEA docking pose Group **2** where the polar head moiety of AEA occupied HC; and 3) AEA docking pose Group **3** where HC was left unoccupied. Three representative poses (*docking pose1*, *docking pose2* and *docking pose3*) were selected from AEA docking pose Group **1**. Similarly, three (*docking pose4*, *docking pose5* and *docking pose6*) and two (*docking pose7* and *docking pose8*) representative poses were selected from AEA docking pose Group **2** and AEA docking pose Group **3**, respectively. Overall, a total of eight docking poses were selected (Fig 2).

### MD simulations of the CB1-Gi assembly

Each of the eight selected AEA docking poses inserted into the binding pocket of the CB1 receptor in the CB1-Gi complex model in a fully hydrated 1-palmitoyl-2-oleoyl-*sn*-glycero-3-phosphocholine (POPC) lipid bilayer was subjected to energy minimization (5,000 iterations). This was followed by an MD simulation at 310 K in the NPT ensemble to obtain an all-atom, solvent-equilibrated AEA binding pose. A long time (typically 200 ns) MD simulation was performed to ascertain that receptor binding interaction of AEA became optimally established as indicated by the root-mean-square deviations (RMSDs) of the receptor as well as the bound ligand AEA. During the MD simulations, the area per lipid was ∼65 Å^2^, which is in agreement with the experimentally measured values of the biologically relevant liquid-crystalline phase [26].

### Simulation protocol

All simulations were performed using the NAMD simulation package (ver. 2.7 Linux-x86_64) [27], using CHARMM36 force field parameters for proteins with the ϕ/ψ angle cross-term map correction [28, 29] and lipids [30], and the TIP3P water model [31]. The temperature was maintained at 310 K through the use of Langevin dynamics [32] with a damping coefficient of 1/ps. The pressure was maintained at 1 atm by using the Nosé-Hoover method [33] with the modifications as described in the NAMD user’s guide. The van der Waals interactions were switched at 10 Å and zero smoothly at 12 Å. Electrostatic interactions were treated using the Particle Mesh Ewald (PME) method [34]. A pair list for calculating the van der Waals and electrostatic interactions was set to 13.5 Å and updated every 10 steps. A multiple time-stepping integration scheme, the impulse-based Verlet-I reversible reference system propagation algorithm method [35], was used to efficiently compute full electrostatics. The time step size for integration of each step of the simulation was 2 fs.

### CHARMM parameterization

To describe AEA in the MD simulations, CHARMM parameters for AEA, compatible with the CHARMM36 all-atom additive force field, were determined manually in consideration of the CHARMM force field parametrization strategy. Thus, to minimize any inconsistency with the existing CHARMM parameters, the parameters for AEA were borrowed from those parameters of moieties with similar chemical properties properly identified from the existing CHARMM36 parameter sets. Because the structure of AEA consists of the arachidonyl moiety and the ethanol amide moiety (see Fig 1), the parameters for the arachidonyl moiety was from the parameters for arachidonic acid (ARAC) (https://www.ks.uiuc.edu/Research/namd/wiki/index.cgi?ArachidonicAcidTop) and the parameters for the ethanol amide moiety was found in the existing CHARMM36 parameter sets. In comparison, the resulting parameters of AEA, shown in S1 File, were almost identical with those generated by the parameter server for CGENFF (http://charmm-gui.org/).

### RMSD analysis

RMSD values of the CB1 receptor were calculated by root mean square fitting to the initial coordinates with respect to the backbone Cα atoms of the transmembrane (TM) helical residues of the CB1 receptor (TM1: Pro113–His143; TM2: Tyr153–His178; TM3: Arg186–Ser217; TM4: Arg230–Val249; TM5: Glu273–Ala301; TM6: Met337–Ile362; and TM7: Lys373–Arg400). The RMSD values of the polar head moiety of AEA bound to the above fitted CB1 receptor were calculated by using the initial coordinates of its heavy atoms as the reference structure (see Fig 1). Similarly, the RMSD values of the hydrophobic tail moiety of AEA bound to the above fitted CB1 receptor were calculated by using the initial coordinates of its heavy atoms as the reference structure (see Fig 1).

### Key binding residue analysis

Key binding pocket residues important for AEA binding were identified by examining the CB1 receptor pocket residues within 4 Å of the bound ligand AEA during the simulation. The analysis was performed for the three parts of AEA (see Fig 1): 1) the head moiety; 2) the polyene linker moiety (C5-C15); and 3) the hydrophobic tail moiety (C16-C20). For the polar head moiety of AEA, the focus was on the polar and the charged residues that form hydrogen bonds. For the polyene linker moiety, the focus was on the aromatic residues that form aromatic-π stacking interactions. For the hydrophobic tail moiety of AEA, the focus was on the hydrophobic residues that form van der Waals interactions. For hydrogen bonds, a criterion of 3 Å between the hydrogen bond donor and acceptor atoms was used. For the aromatic-π stacking, a criterion of 6 Å between the center of the double bond of AEA and the centroid of an aromatic ring was used.

### AEA non-bonding interaction energies

NAMD Energy Plugin as implemented in VMD [36] was used to calculate the AEA non-bonding interaction energy values. A smooth switching function was activated at the distance of 10 Å to truncate the non-bonding interaction energies smoothly at the cutoff distance of 12 Å. The energy values with the standard deviation of the values in parentheses were averaged over the last 25 ns of the simulation.

To better estimate the AEA non-bonding interaction energy values, quantum mechanics methods were also used. The binding pocket residues, water molecules and lipids within 3.6 Å of the bound ligand were extracted from over the last 25 ns of the simulation. From these extracted coordinates an average structure was calculated. The N-terminus and C-terminus of each of the binding pocket residues were capped by adding an acetyl group and an N-methyl group, respectively. The resulting structure was subjected to a short energy minimization of 2,500 iterations using the CHARMM36 all-atom force field, during which the residue backbone atoms were fixed. The semiempirical PM3 method [37] and the density functional theory (DFT) M06-2X method [38] with the 6-31G* basis set, as implemented in Spartan’18 (Wavefunction, Inc. Irvine, CA), were used to calculate the AEA non-bonding interaction energy values.

## Results

### Eight representative AEA docking poses selected from AutoDock docking runs

The eight representative docking poses selected from more than one hundred AutoDock docking runs are shown in Fig 2. These AEA docking poses were clustered into AEA docking pose Group **1**, AEA docking pose Group **2** and AEA docking pose Group **3**, depending upon how AEA interacted with HC. In our AutoDock docking runs, AEA sometimes occupied HC deeper than AM11542 in the X-ray crystal structure of AM11542-bound CB1 receptor [6]. Thus, this extended, deep HC was called HC_d_.

In the first three selected AEA docking poses (named *docking pose1*, *docking pose2* and *docking pose3*) that belonged to AEA docking pose Group **1**, the tail moiety of AEA commonly occupied HC or HC_d_ (Fig 2A). *Docking pose1*, where the head moiety of AEA bound the H7 region and the tail moiety of AEA occupied HC_d_, was assigned to be docking pose **1_H7_HC**_**d**_ (“**1**” denotes Group **1**, “**H7**” denotes the H7 region where the head moiety binds, and “**HC**_**d**_” denotes HC_d_ where the tail moiety binds). Similarly, *docking pose2*, where the head moiety bound the H2/H3 region and the tail moiety occupied HC_d_, was assigned to be docking pose **1_H2/H3_HC**_**d**_. *Docking pose3*, where the head moiety bound the H7 region and the tail moiety occupied HC, was assigned to be docking pose **1_H7_HC**.

For the next three selected AEA docking poses (named *docking pose4*, *docking pose5* and *docking pose6*) that belonged to pose Group **2**, the head moiety of AEA commonly occupied HC or HC_d_ (Fig 2B). *Docking pose4*, where the head moiety of AEA occupied HC_d_ and the tail moiety bound the H2/H3 region, was assigned to be docking pose **2_HC**_**d**_**_H2/H3** (“**2**” denotes Group **2**, “**HC**_**d**_” denotes HC_d_ where the head moiety binds, and “**H2/H3**” denotes the H2/H3 region where the tail moiety binds). *Docking pose5*, where the head moiety occupied HC_d_ and the tail moiety bound the H7 region, was assigned to be docking pose **2_HC**_**d**_**_H7**. *Docking pose6*, where the head moiety occupied HC and the tail moiety pointed toward the pocket outer core region, was assigned to be docking pose **2_HC_OC** (“**OC**” denotes the ***o****uter* ***c****ore* region).

For the last two selected AEA docking poses (named *docking pose7* and *docking pose8*) that belonged to pose Group **3**, HC was commonly left unoccupied (Fig 2C). *Docking pose7*, where the head moiety pointed toward the pocket inner core region and the tail moiety bound the H2/H3 region, was assigned to be docking pose **3_IC_H2/H3** (“**3**” denotes Group **3**, “**IC**” denotes the ***i****nner* ***c****ore* region where the head moiety binds and “**H2/H3**” denotes the H2/H3 region where the tail moiety binds). *Docking pose8*, where the head moiety bound the H7 region and the tail moiety bound the pocket inner core, was assigned to be docking pose **3_H7_IC**. These eight representative docking poses are summarized in Table 1.

**Table 1:** Receptor interactions of the eight docking poses selected from AutoDock runs and the corresponding equilibrated poses in simulation.

AEA pose	AEA—CB1 binding pocket interactions				Pose group
	H2/H3 region	H7 region	Hydrophobic channel (HC)	Deep hydrophobic channel (HC_d_)	
*Docking pose1*		head		tail	**1_H7_HC**_**d**_
*Equilibrated pose1*^a^^)^		head	tail		**1_H7_HC**
*Docking pose2*	head			tail	**1_H2/H3_HC**_**d**_
*Equilibrated pose2*^a^^)^	head		tail		**1_H2/H3_HC**
*Docking pose3*		head	tail		**1_H7_HC**
*Equilibrated pose3*^a^^)^		head	tail		**1_H7_HC**
*Docking pose4*	tail			head	**2_HC**_**d**_**_H2/H3**
*Equilibrated pose4*^a^^)^	tail			head	**2_HC**_**d**_**_H2/H3**
*Docking pose5*		tail		head	**2_HC**_**d**_**_H7**
*Equilibrated pose5*^a^^)^	tail			head	**2_HC**_**d**_**_H2/H3**
*Docking pose6*			head		**2_HC_OC**
*Equilibrated pose6*^a^^)^	tail			head	**2_HC**_**d**_**_H2/H3**
*Docking pose7*	tail				**3_IC_ H2/H3**
*Equilibrated pose7*^a^^)^	tail			head	**2_HC**_**d**_**_H2/H3**
*Docking pose8*		head			**3_H7_IC**
*Equilibrated pose8*^a^^)^		head	tail		**1_H7_HC**

^a^Docking pose in simulation.

### RMSD analysis of the eight representative AEA docking poses

**Fig 3:**
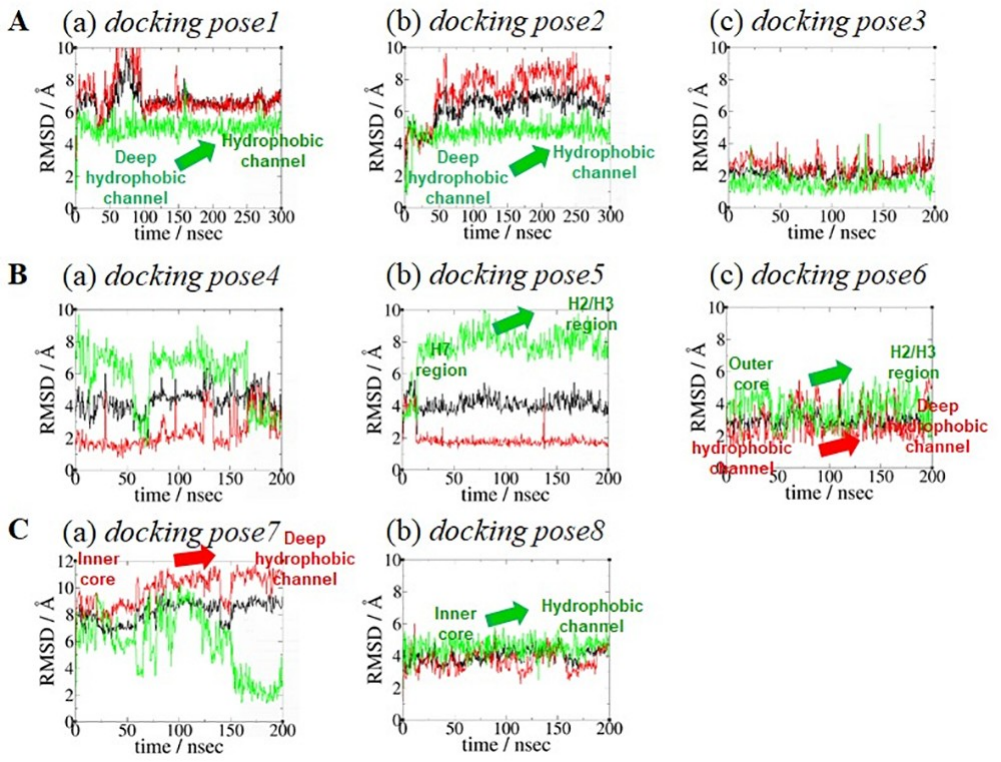
RMSD plots for AEA in the eight AEA docking poses. (A) AEA docking pose Group **1** (*docking pose1*, *docking pose2* and *docking pose3*). (B) AEA docking pose Group **2** (*docking pose4*, *docking pose5* and *docking pose6*). (C) AEA docking pose Group **3** (*docking pose7* and *docking pose8*). The RMSD values of the whole molecule (in black), the polar head moiety (in red) and the hydrophobic tail moiety (in green) of the bound AEA in eight AEA docking poses were calculated with respect to the initial coordinates (heavy atoms only) after fitting the proteins based upon the backbone heavy atoms of the TM helical residues of the CB1 receptor.

As shown in S2 Fig, most of the receptors in the AEA-bound CB1-Gi complex model systems were converged with the RMSD values < 2 Å with respect to the Cα atoms of the receptor TM helical bundle, indicating the CB1 receptor models became stable at the end of the simulation. Similarly, all the ligands in the AEA-bound CB1-Gi complex model systems were converged at the end of the simulation (Fig 3), indicating the bound ligand became stable. The RMSD values of the head moiety and the tail moiety of the bound ligand in the eight AEA docking poses are also shown in Fig 3. Significant increases in the RMSD values are seen for the hydrophobic tail moiety of AEA in *docking pose1* and *docking pose2* (a shift out from HC_d_ to HC), *docking pose5* and *docking pose6* (due to a shift from the H7 or OC region to the H2/H3 region) and *docking pose8* (due to a shift from the IC region to HC) (Fig 3). Similarly, significant increases in the RMSD values are seen for the polar head moiety of AEA in *docking pose6* (due to a shift from HC to HC_d_), *docking pose7* (due to a shift from the binding pocket inner core region to HC_d_) (Fig 3). The eight equilibrated poses (i.e., docking poses in simulation) are summarized in Table 1.

### Three AEA binding poses merged from eight equilibrated poses

**Fig 4:**
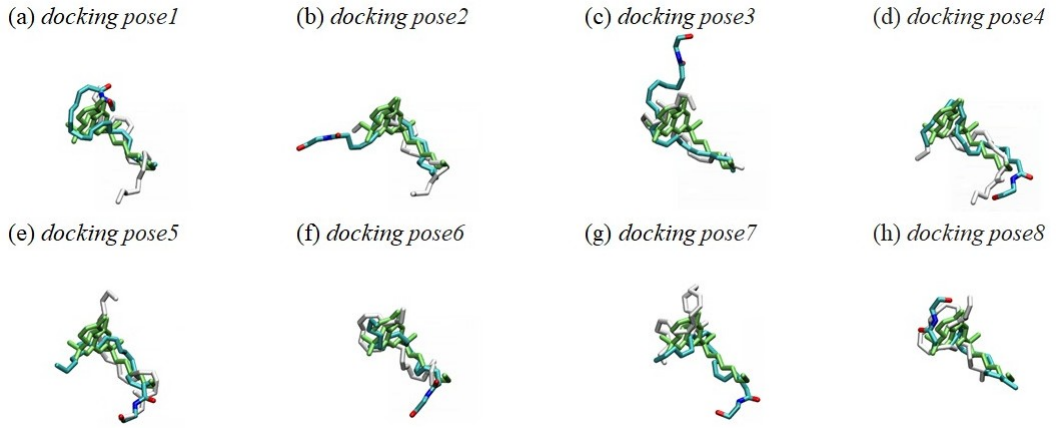
Overlay of the eight docking poses and equilibrated poses of AEA. The eight docking poses (in white) and equilibrated poses (in atom type) of AEA were superimposed to AM11542 (in green) in the X-ray crystal structure of AM11542-bound CB1 receptor [6]. The alignment rule: the backbone Cα atoms of the TM helical residues of the CB1 receptor. Color coding: carbon, cyan; oxygen, red; and nitrogen, blue. Hydrogen atoms were omitted for clarity.

Most of the AEA equilibrated poses are quite different from their initial docking poses in conformation and position (Fig 4), as seen in the RMSD plots of these AEA docking poses (Fig 3).

**Fig 5:**
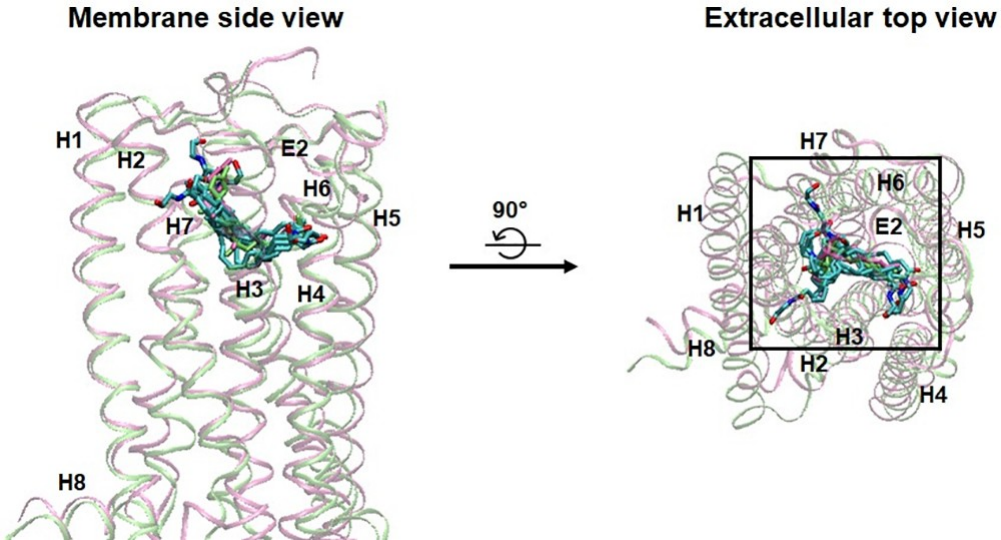
Overlay of eight AEA equilibrated poses and the bound ligands in the X-ray crystal structures of the CB1 receptor. All the eight AEA equilibrated poses (in atom type) were overlaid to AM11542 (in green) and CP55940 (in mauve) after the receptors in these poses were superimposed to the receptors in the X-ray crystal structures of the AM11542-bound CB1 receptor [6] and the CP55940-bound CB1 receptor [7]. The alignment rule of the receptors was same as in Fig 4. Color coding: carbon, cyan; oxygen, red; and nitrogen, blue. Hydrogen atoms were omitted for clarity.

As shown in Fig 5, the AEA equilibrated poses share the binding region in the orthosteric binding pocket well with the bound cannabinoid ligands as found in the X-ray crystal structures of the CB1 receptor [6, 7]. Each of the eight AEA equilibrated poses adopts an extended conformation that spans throughout the binding core region.

**Fig 6:**
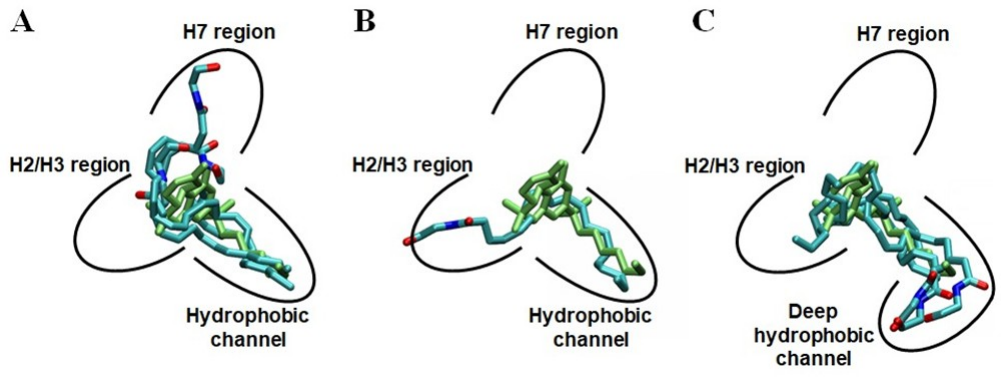
Three AEA binding poses. (A) AEA binding pose **1_H7_HC** (*equilibrated pose1*, *equilibrated pose3* and *equilibrated pose8*). (B) AEA binding pose **1_H2/H3_HC** (*equilibrated pose2*). (C) AEA binding pose **2_HC**_**d**_**_H2/H3** (*equilibrated pose4*, *equilibrated pose5*, *equilibrated* pose6 and *equilibrated pose7*).

After overlaid to the bound cannabinoid ligands in the orthosteric binding pocket as found in the X-ray crystal structures of the CB1 receptor [6, 7], the eight AEA equilibrated poses became merged into three AEA binding poses: **1_H7_HC** (*equilibrated pose1*, *equilibrated pose3* and *equilibrated pose8*) (Fig 6A); **1_H2/H3_HC** (*equilibrated pose2*) (Fig 6B); and **2_HC**_**d**_**_H2/H3** (*equilibrated pose4*, *equilibrated pose5*, *equilibrated pose6* and *equilibrated pose7*) (Fig 6C). In these binding poses, HC was always occupied by either the tail moiety or the head moiety of AEA, suggesting that HC is essential for AEA binding.

### Key binding residue analysis of three AEA binding poses

**Fig 7:**
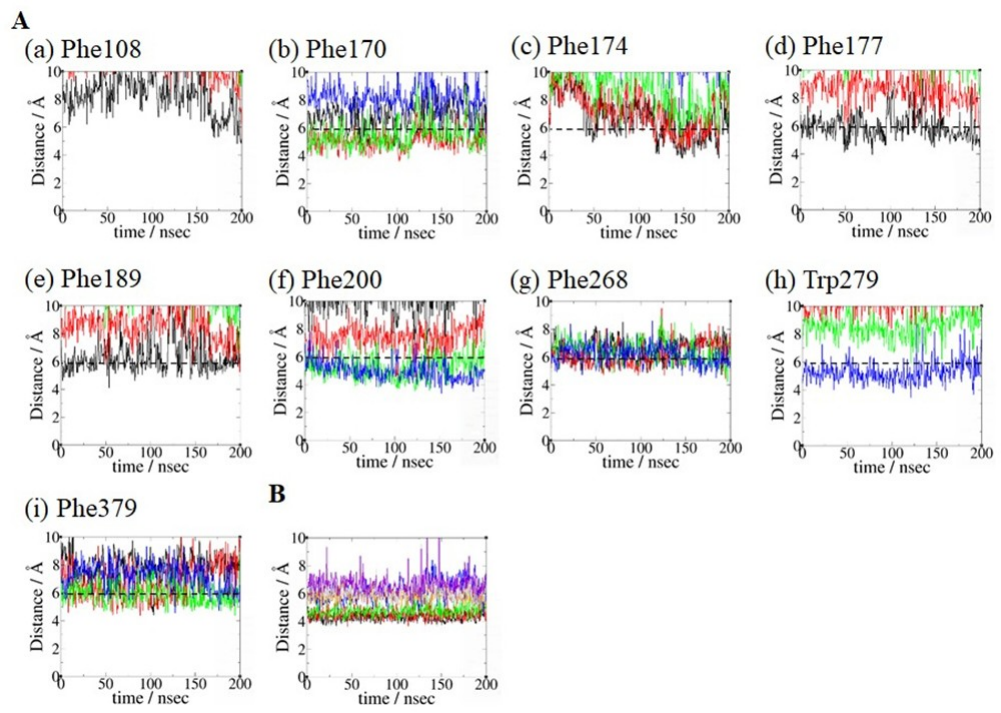
Key binding residue analysis of AEA binding pose 1_H7_HC. (A) Interactions with the polyene linker moiety of AEA. Interactions between the four double bonds with (a) Phe108, (b) Phe170, (c) Phe174, (d) Phe177, (e) Phe189, (f) Phe200, (g) Phe268, (h) Trp279 and (i) Phe379. The distances between the aromatic ring centroid of the aromatic side chain and the centers of the first double bond (C5 = C6) (in black), the second double bond (C8 = C9) (in red), the third double bond (C11 = C12) (in green) and the fourth double bond (C14 = C15) (in blue) of AEA. Aromatic ring centroid distance of 6 Å (in dotted line) was used to approximately assess the π-aromatic interactions. (B) Interactions with the hydrophobic tail moiety of AEA. The distances between the center of mass of the terminal alkyl moiety (C17-C20) of AEA and the centers of mass of the side chains of Thr197 (in black), Trp279 (in red), Phe268 (in green), Leu193 (in blue), Met363 (in orange), Leu276 (in brown), Ile271 (in grey) and Tyr275 (in violet).

**Fig 8:**
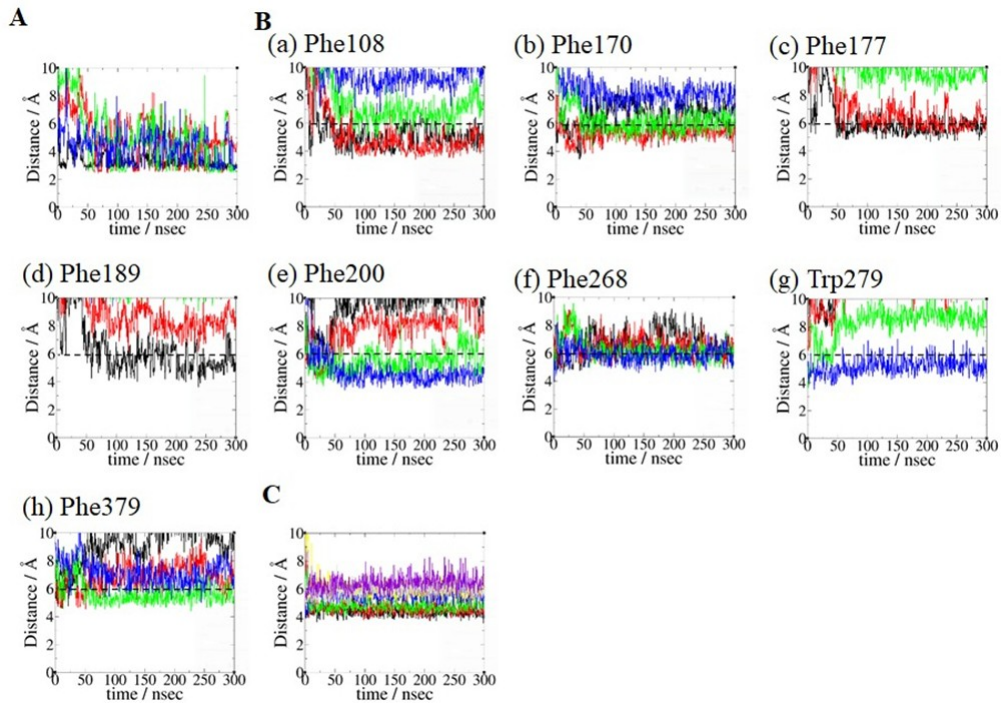
Key binding residue analysis of AEA binding pose 1_H2/H3_HC. (A) Interactions with the head moiety of AEA. The hydrogen bonding distance between the amide nitrogen atom of the head moiety of AEA and the side chain oxygen atom of Ser173 (in black). The hydrogen bonding distances between the hydroxyl oxygen atom of the head moiety of AEA and two side chain oxygen atoms O_δ1_ (in red) and O_δ2_ (in green) of Asp176. The hydrogen bonding distance between the hydroxyl oxygen atom of the head moiety of AEA and the side chain nitrogen atom of Lys192 (in blue). (B) Interactions with the polyene linker moiety of AEA. Interactions between the four double bonds with (a) Phe108, (b) Phe170, (c) Phe177, (d) Phe189, (e) Phe200, (f) Phe268, (g) Trp279 and (h) Phe379. The criterion for assessing the π-aromatic interactions is same as in Fig 7A. Color coding for the aromatic centroid distances is same as in Fig 7A. (C) Interactions with the hydrophobic tail moiety of AEA. The distances between the center of mass of the terminal alkyl moiety (C17-C20) of AEA and the center of mass of the side chain of Thr197 (in black), Trp279 (in red), Phe268 (in green), Leu193 (in blue), Met363 (in orange), Leu276 (in brown), Ile271 (in grey) and Tyr275 (in violet).

**Fig 9:**
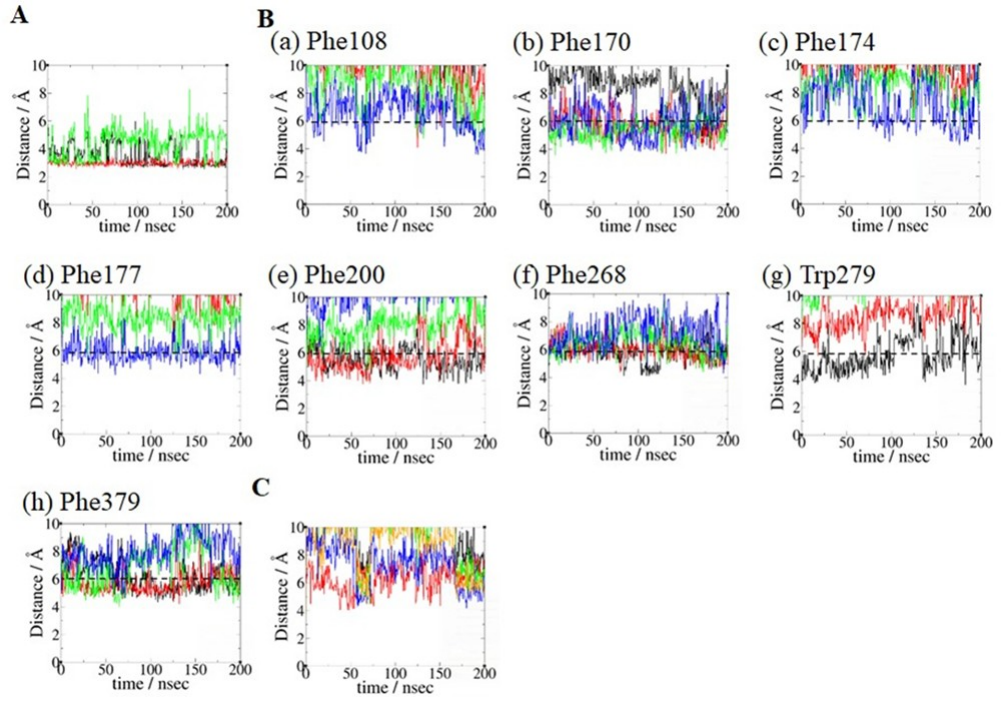
Key binding residue analysis of AEA binding pose 2_HC_d__H2/H3. (A) Interactions with the head moiety of AEA. The hydrogen bonding distances between the hydroxyl oxygen atom of the head moiety of AEA and the side chain oxygen atoms of Thr197 (in black) and Tyr275 (in green). The hydrogen bonding distance between the amide nitrogen atom of the head moiety of AEA and the side chain oxygen atom of Thr197 (in red). (B) Interactions with the polyene linker moiety of AEA. Interactions between the four double bonds with (a) Phe108, (b) Phe170, (c) Phe174, (d) Phe177, (e) Phe200, (f) Phe268, (g) Trp279 and (h) Phe379. The criterion for assessing the π-aromatic interactions is same as in Fig 7A. Color coding for the aromatic centroid distances is same as in Fig 7A. (C) Interactions with the hydrophobic tail moiety of AEA. The distances between the center of mass of the terminal alkyl moiety (C17-C20) of AEA and the center of mass of the side chain of Ile169 (in black), Phe177 (in red), Lys192 (in green), Leu193 (in blue) and Val196 (in orange).

The results of the key binding residue analysis of AEA binding poses **1_H7_HC** (Fig 7) **1_H2/H3_HC** (Fig 8) and **2_HC**_**d**_**_H2/H3** (Fig 9) are described below. For the binding pose **1_H7_HC**, *equilibrated pose3* was used. For the binding pose **1_H2/H3_HC**, *equilibrated pose2* was used. For the binding pose **2_HC**_**d**_**_H2/H3**, *equilibrated pose4* was used.

a) AEA equilibrated pose **1_H7_HC**. No noticeable hydrogen bond involving the head moiety of AEA was observed. Because our focus was on the aromatic residues that form aromatic-π stacking interactions with the polyene linker moiety of AEA, the aromatic residues responsible for interacting with the four double bonds in AEA were identified (Fig 7A): Phe108, Phe174, Phe177 and Phe189 with the first double bond (C5 = C6) of AEA; Phe108, Phe170 and Phe268 with the second double bond (C8 = C9) of AEA; Phe170, Phe200, Phe268 and Phe379 with the third double bond (C11 = C12) of AEA; and Phe200, Phe268, Trp279 and Phe379 with the fourth double bond (C14 = C15). These aromatic-π stacking interactions of the polyene linker moiety of AEA are shown in S3A Fig. As shown in Fig 7B, key binding residues that interacted with the hydrophobic tail moiety of AEA include Leu193, Thr197, Phe268, Ile271, Tyr275, Leu276, Trp279 and Met363 that remained quite close to the terminal alkyl moiety (C17-C20) of AEA throughout the simulation. Most of these residues were identified as the HC-forming residues in the X-ray crystal structure of the AM11542-bound CB1 receptor [6].
b) AEA binding pose **1_H2/H3_HC**. The hydroxyl oxygen atom of the head moiety of AEA formed hydrogen bonds to both Asp176 of H2 and Lys192 of H3, while the amide nitrogen atom of the head moiety of AEA formed a hydrogen bond to Ser173 of H2 (Fig 8A). These hydrogen bonds remained stable in the later stage of the simulation. Lys192 of H3 also formed a salt bridge to Asp184, which formed a water-mediated hydrogen bond to Asp176 (S4 Fig). As shown in Fig 8B, the aromatic residues that interacted closely with the polyene linker moiety of AEA were identified: Phe108, Phe177, Phe189 and Phe268 with the first double bond (C5 = C6) of AEA; Phe108, Phe170, Phe177 and Phe268 with the second double bond (C8 = C9) of AEA; Phe170, Phe200, Phe268 and Phe379 with the third double bond (C11 = C12) of AEA; and Phe200, Phe268, Trp279 and Phe379 with the fourth double bond (C14 = C15) of AEA. These aromatic-π stacking interactions of the polyene linker moiety of AEA are shown in S3B Fig. It is worth noting that Phe268 interacted with all the double bonds of the polyene linker moiety (Fig 8B(f)). As shown in Fig 8C, the HC-forming residues, same as those in the binding pose **1_H7_HC**, remained quite close to the terminal alkyl moiety (C17-C20) of AEA throughout the simulation.
c) AEA binding pose Group **2_HC**_**d**_**_H2/H3**. The hydroxyl oxygen atom of the head moiety of AEA formed a hydrogen bond to Thr197 and alternatively to Tyr275 (Fig 9A). The amide nitrogen atom of the head moiety of AEA formed another hydrogen bond to Thr197, which remained stable throughout the simulation. As shown in Fig 9B(a) through (h), the aromatic residues that interacted closely with the polyene linker moiety of AEA were identified: Phe200, Phe268, Trp279 and Phe379 with the first double bond (C5 = C6) of AEA; Phe108, Phe170, Phe200, Phe268 and Phe379 with the second double bond (C8 = C9) of AEA; Phe108, Phe170, Phe268 and Phe379 with the third double bond (C11 = C12); and Phe108, Phe170, Phe174 and Phe177 with the fourth double bond (C14 = C15). These aromatic-π stacking interactions of the polyene linker moiety of AEA are shown in S3C Fig. As shown in Fig 9C, Ile169, Phe177, Lys192, Leu193 and Val196 remained close to the terminal alkyl moiety (C17-C20) of AEA in the later stage of the simulation.

## Discussion

### Key structural features of three AEA binding poses

**Fig 10:**
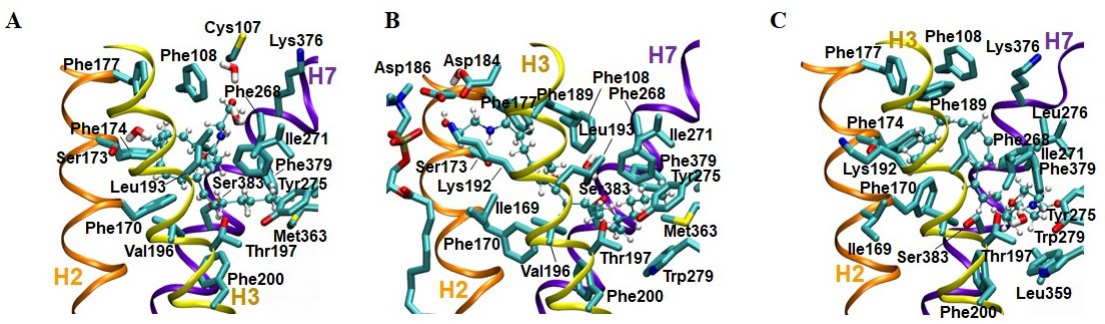
Key binding pocket residues in three AEA binding poses. (A) AEA binding pose 1_H7_HC. (B) AEA binding pose 1_H2/H3_HC. (C) AEA binding pose 2_HC_d__H2/H3.

Key structural features of each of three AEA binding poses are described below:

a) AEA binding pose **1_H7_HC**. The key receptor interactions of AEA are shown in Fig 10A. The hydrophobic tail moiety was well aligned with the DMH tails of the bound AM11542 and CP55940 in the X-ray crystal structures of the CB1 receptor [6, 7] and the head moiety bound the H7 region. It is interesting to see that not only the terminal five carbons (C16-C20) but also the fourth double bond (C14 = C15) bound HC.
b) AEA binding pose **1_H2/H3_HC**. The key receptor interactions of AEA are shown in Fig 10B. The tail moiety occupied HC just as in the binding pose **1_H7_HC**, while the head moiety bound the H2/H3 region. The head moiety bound the H2/H3 region under E1 extensively (see S4 Fig). It appears that the extensive H-bonding and salt bridge network centered at Lys192 contributes favorably to the binding of the polar head moiety of AEA. The head moiety of AEA also interacted with a lipid molecule through the hydrogen bond between the hydroxyl oxygen atom of the head moiety of AEA and the phosphate oxygen atom of the polar head group of the lipid (see S4 Fig).
c) AEA binding pose **2_HC**_**d**_**_H2/H3**. The detailed receptor interactions of AEA are shown in Fig 10C. In this binding pose, the polar head moiety bound to HC_d_ while the tail moiety bound to the H2/H3 region. The binding pocket residues that interacted with the tail moiety of AEA, though limited in extent, are somewhat similar to those that interacted with the tail moiety of AEA in the binding pose **1_H2/H3_HC**. The binding pocket residues that interacted with the head moiety are like those in the binding pose **1_H7_HC** or **1_H2/H3_HC**. However, the terminal hydroxyethyl group of the head moiety occupied HC_d_. It appears that the H-bonds between the polar head group and the polar residues play key roles in stabilizing the polar head moiety deep inside HC.

### Importance of the hydrophobic channel in AEA binding to the CB1 receptor

All the eight representative AEA equilibrated poses showed that AEA interacted tightly with HC (see Fig 6). If either the tail moiety or the head moiety initially occupied HC, it remained there throughout the simulation. However, if HC was initially left empty, it became occupied by either the tail moiety or the head moiety in simulation. These results underscore the importance of HC of the CB1 receptor in AEA binding just as seen in the recent X-ray crystal structures of the CB1 receptor in complex with various ligands [4, 6, 7, 39]. In support, it has been reported that Ala mutations of the HC-forming residues Leu193 and Met363 of the CB1 receptor caused ~80-fold and ~4-fold decreases, respectively, in CP55940 binding [40]. Since the tail moiety of AEA in the binding poses **1_H7_HC** and **1_H2/H3_HC** was exactly overlaid to the DMH tail of AM11542 [6] and CP55940 [7], mutation of these hydrophobic pocket residues would also alter AEA binding affinity.

It is surprising to see in the present study that the polar head moiety of AEA was also able to stably occupy HC_d_ as in the binding pose **2_HC**_**d**_**_H2/H3** (Fig 6C). It appears that the stabilization of the polar head moiety of AEA through H-bonding is required for its binding to HC_d_.

### Which binding region is a privileged subsite?

It is shown from the present study that regardless of whether the hydrophobic pocket was occupied or empty in the initial docking poses, HC became preferentially occupied in all of the eight equilibrated poses. Therefore, it is likely that if the one end moiety (either the head moiety or the tail moiety) of AEA establishes its binding interaction with HC as the primary binding contact, then the other end moiety of AEA establishes its binding to either the H2/H3 region or the H7 region before the conformationally flexible linker moiety completes AEA binding to the receptor.

The recently determined X-ray crystal structure of the classical cannabinoid agonist AM11542-bound CB1 receptor [6] reveals that the trimethyl substituted B/C-ring moiety of AM11542 binds the H2/H3 region. Similarly, the X-ray crystal structure of the nonclassical CP55940-bound CB1 receptor [7] reveals that the propylhydroxyl substituted C-ring moiety of CP55940 binds preferentially the H2/H3 region. A 10-fold increase in binding affinity by the introduction of the propylhydroxyl group to the C-ring of CP47497, which becomes equivalent to CP55940 [41], supports the idea that the H2/H3 region is important for cannabinoid binding. Alanine mutations of the H2/H3 residues Phe174, Phe177, Asp184, Phe189, Lys192 and Leu193 resulted in significant decreases in binding affinity of CP55940 [8, 40, 42, 43], also underscoring the importance of the H2/H3 region in cannabinoid binding. Collectively, these experimental results indicate that the H2/H3 region of the CB1 receptor is a ligand binding subsite privileged over the H7 region.

In the present study, AEA interacted with the H2/H3 region in the binding poses **1_H2/H3_HC** (i.e., the head moiety) (Fig 6B) and **2_HC**_**d**_**_H2/H3** (i.e., the tail moiety) (Fig 6C), while AEA little interacted with the H2/H3 region in the binding pose **1_H7_HC** (Fig 6A). The binding pose **1_H2/H3_HC** uniquely showed extensive H2/H3 interactions of the head moiety of the ligand (see S4 Fig). As shown in Table 2 and S1–S3 Tables, the CB1-AEA binding interactions were estimated by the non-bonding interaction energy between the binding pocket residues and the bound ligand AEA by using both molecular mechanics CHARMM36 force field [28] and quantum mechanics semiempirical PM3 [37] and DFT M06-2X [38] methods. Based on the estimated non-bonding interaction energy values, it is predicted that the binding pose **1_H2/H3_HC** can interact with the receptor more strongly than the binding pose **1_H7_HC**. Because the tail moiety of the ligand interacted with HC quite similarly in these binding poses (see Figs 6 and 10), the favorable binding interactions shown in the binding pose **1_H2/H3_HC** over the binding pose **1_H7_HC** suggests that AEA interactions with the H2/H3 region are more important than with the H7 region. Based on the estimated non-bonding interaction energy values, it is also predicted that the binding pose **2_HC**_**d**_**_H2/H3** can interact with the receptor as an alternative pose, though exhibiting slightly decreased binding, to the binding poses **1_H7_HC** and **1_H2/H3_HC**.

**Table 2:** Non-bonding interaction energies of AEA binding poses 1_H7_HC, 1_H2/H3_HC and 2_HC_d__H2/H3 calculated by using CHARMM36 force field [28], PM3 [37] and DFT M06-2X [38] methods.

	CHARMM36^a^^)^	PM3^b^^)^	M06-2X/6-31G*^b^^)^
AEA binding pose **1_H7_HC**			
Equilibrated *pose1*	-71.97	-29.35	-66.79
Equilibrated *pose3*	-81.88	-33.98	-81.65
Equilibrated *pose8*	-84.57	-29.27	-72.93
AEA binding pose **1_H2/H3_HC**			
Equilibrated *pose2*	-91.22	-36.75	-95.95
Equilibrated *pose2’*	-76.38	-36.39	-76.70
AEA binding pose **2_HC**_**d**_**_H2/H3**			
Equilibrated *pose4*	-75.37	-24.62	-68.87
Equilibrated *pose5*	-77.01	-28.85	-78.36
Equilibrated *pose6*	-72.59	-25.09	-71.06
Equilibrated *pose7*	-78.00	-28.40	-74.29

^a^By using NAMD Energy Plugin as implemented in VMD [36].

^b^As implemented in Spartan’18 (Wavefunction, Inc. Irvine, CA).

### Which AEA binding pose is the best candidate for the bioactive conformation?

If we assume that all the binding poses **1_H7_HC**, **1_H2/H3_HC** and **2_HC**_**d**_**_H2/H3** as potential candidates for the bioactive conformation, the measured binding affinity of AEA to the CB1 receptor would be the results of the binding of these poses in equilibrium. If the binding poses **1_H7_HC** and **2_HC**_**d**_**_H2/H3** are weaker binding modes than the binding pose **1_H2/H3_HC**, as predicted by the estimated the CB1-AEA binding interaction (Table 2), some ligand binding interactions exerted by the binding poses **1_H7_HC** and **2_HC**_**d**_**_H2/H3** may still be present but would be weaker than those exerted by the binding pose **1_H2/H3_HC**. In this regard, an increase in CB1 binding affinity by substituting the 2-hydroxyethyl group of AEA with a cyclopropyl ring or a halogen [44, 45] is intriguing. It is possible that such a hydrophobic substitution for the polar head moiety of AEA would depreciate the binding pose **2_HC**_**d**_**_H2/H3**, a binding pose weaker than the binding pose **1_H2/H3_HC**, contributing to an increase in AEA binding affinity overall.

Compared with the binding pose **2_HC**_**d**_**_H2/H3**, the binding pose **1_H2/H3_HC** exhibits extensive binding interactions with the H2/H3 region under E1, including H-bonds to Asp176 and Lys192 (S4 Fig). On the other hand, the binding interactions with HC in the binding pose **1_H2/H3_HC** (Fig 10B) are presumably less extensive than the binding interactions with HC_d_ in the binding pose **2_HC**_**d**_**_H2/H3** (Fig 10C). Therefore, it is expected that the overall binding interactions in the binding poses **1_H2/H3_HC** and **2_HC**_**d**_**_H2/H3** would be quite competitive. However, the estimated CB1-AEA binding interaction energy values predict that the binding pose **1_H2/H3_HC** binds the receptor stronger than the binding pose **2_HC**_**d**_**_H2/H3** (Table 2), suggesting that the binding interactions of the polar head moiety with HC_d_ in the binding pose **2_HC**_**d**_**_H2/H3** is not advantageous for compensating for its limited binding interactions of the tail moiety with the H2/H3 region. Moreover, the binding pose **2_HC**_**d**_**_H2/H3** would not be a plausible binding mode in physiological environments, because the polar head moiety of AEA would not easily reach HC located deep inside the binding pocket.

On the other hand, the binding pose **1_H2/H3_HC** could be a better candidate for the bioactive conformation than the binding pose **1_H7_HC**, in consideration of the recent X-ray crystal structures of the CB1 receptor [6, 7] and the available mutational data [8, 40, 42, 43] that suggest the H2/H3 region of the CB1 receptor offers a binding subsite privileged over the H7 region. Overall, the binding pose **1_H2/H3_HC** could be the best candidate for the bioactive conformation of AEA at the CB1 receptor. This conclusion agrees with a recent study of the binding mode predictions of various AEA-like endocannabinoids guided by molecular mechanics-Poisson-Boltzmann surface area (MM-PBSA) binding free energy calculations [46]. The AEA binding mode in this study is quite similar to our AEA binding pose **1_H2/H3_HC**. To check the validity of the binding pose **1_H2/H3_HC**, we carried out another independent MD simulation, starting from a docking pose (named *docking pose2’*) different from *docking pose2* within AEA docking pose group **1_H2/H3_HC** (S5A Fig). The resulting *equilibrated pose2’* was quite similar to *equilibrated pose2* (S5B Fig). Its estimated CB1-AEA binding interaction energy value, however, was not as low as that of *equilibrated pose2* (Table 2). Since *equilibrated pose2* showed unique binding interactions with a lipid (S4 Fig), it is possible that AEA-lipid interactions are important for AEA binding to the CB1 receptor.

The chance of the bioactive conformation being present in AEA is much lower than in AM11542 and CP55940, simply because it is difficult for the highly flexible AEA to be locked into the active conformation required for best fitting to the binding pocket. Both the varying polar head moiety and the varying hydrophobic tail of AEA would interfere significantly from achieving the bioactive conformation. Overall, AEA is expected to achieve the active conformation much more laboriously than AM11542 and CP55940, which is possibly related to its known partial agonistic activity [1].

## Conclusions

In summary, we have explored possible binding conformations of AEA within the binding pocket of the CB1 receptor well defined in the recently determined X-ray crystal structures of the ligand-bound CB1 receptors, by using a combination of docking and MD simulation approaches. Because the challenging problem of conformational explosion in AEA structure was significantly reduced owing to the binding preference of AEA to HC, we were able to identify three candidate AEA binding poses for the bioactive conformation at the CB1 receptor. Although the present study was rather limited in exploring all the available binding conformations allowed for the extremely flexible AEA, our results suggest that CB1 receptor interactions of the H2/H3 region as well as HC are important for AEA binding.

## Supporting information

S1 Fig Overlay of HU210 and AM11542. HU210 (in gray) in the binding pocket of the CB1-Gi complex model [23], refined according to the X-ray crystal structure of the AM11542-bound CB1 receptor [6], is overlaid to AM11542 (in green) in the X-ray crystal structure of the AM11542-bound CB1 receptor [6]. The binding pocket residues within 4 Å of the ligand are also displayed.
(PDF)

S2 Fig The RMSD values of the CB1 receptor in the eight AEA docking poses. The RMSD values were calculated by root mean square fitting to the initial coordinates with respect to the backbone heavy atoms of the TM helical residues of the CB1 receptor. (A) AEA docking pose Group **1**. (B) AEA docking pose Group **2**. (C) AEA docking pose Group **3**.
(PDF)

S3 Fig Aromatic-π stacking interactions of the polyene linker moiety of AEA. (A) AEA binding pose **1_H7_HC**. (B) AEA binding pose **1_H2/H3_HC**. (C) AEA binding pose **2_HC**_**d**_**_H2/H3**. The aromatic-π stacking interactions are shown in the dotted lines. A criterion of 6 Å was used to approximate aromatic-π stacking interactions between the centroid of an aromatic ring and the centers of mass of the first double bond (τ_1_, C5 = C6) of AEA (in black), the second double bond (τ_2_, C8 = C9) of AEA (in red), the third double bond (τ_3_, C11 = C12) of AEA (in green) and the fourth double bond (τ_4_, C14 = C15) of AEA (in blue).
(PDF)

S4 Fig Key receptor interactions of the polar head moiety of AEA in AEA binding pose 1_H2/H3_HC. Hydrogen bonding interactions are shown in red dotted lines. Hydrogen bonding distance (in Å) is also shown. Residues and water molecules are shown in stick mode and AEA are shown in space-filling mode.
(PDF)

S5 Fig Analysis of docking pose2’. (A) *Docking pose2’* (in dark green) and the AEA docking poses (in line mode) that belong to the same cluster as *docking pose2* (in pink). (B) AEA binding pose **1_H2/H3_HC** (*equilibrated pose2* and *equilibrated pose2’*) overlaid to AM11542 (in green) and CP55940 (in mauve) in the X-ray crystal structures of the AM11542-bound CB1 receptor [6] and the CP55940-bound CB1 receptor [7]. (C) The RMSD values of the CB1 receptor in *docking pose2’*. (D) The RMSD plots of the head moiety and the tail moiety of AEA in *docking pose2’*. The RMSD values of the polar head moiety (in red) and the hydrophobic tail moiety (in green) of the bound AEA (in red).
(PDF)

S1 Table Non-bonding interaction energies (in kcal/mol) calculated by CHARMM36 all-atom force field [28]. (PDF)

S2 Table Non-bonding interaction energies (in kcal/mol) calculated by the semiempirical PM3 method [37]. (PDF)

S3 Table Non-bonding interaction energies (in kcal/mol) calculated by the DFT M06-2X method [38] with the 6-31G* basis set. (PDF)

S1 File CHARMM parameters for anandamide (AEA). (PDF)

S2 File Topology definitions for anandamide (AEA). (PDF)
