# Supplementary figures and images for: Prediction of essential binding domains for the endocannabinoid N-arachidonoylethanolamine (AEA) in the brain cannabinoid CB1 receptor
Source: PLoS One. 2021 Jun 28;16(6):e0229879. doi: 10.1371/journal.pone.0229879 (PMC8238219; doi:10.1371/journal.pone.0229879)

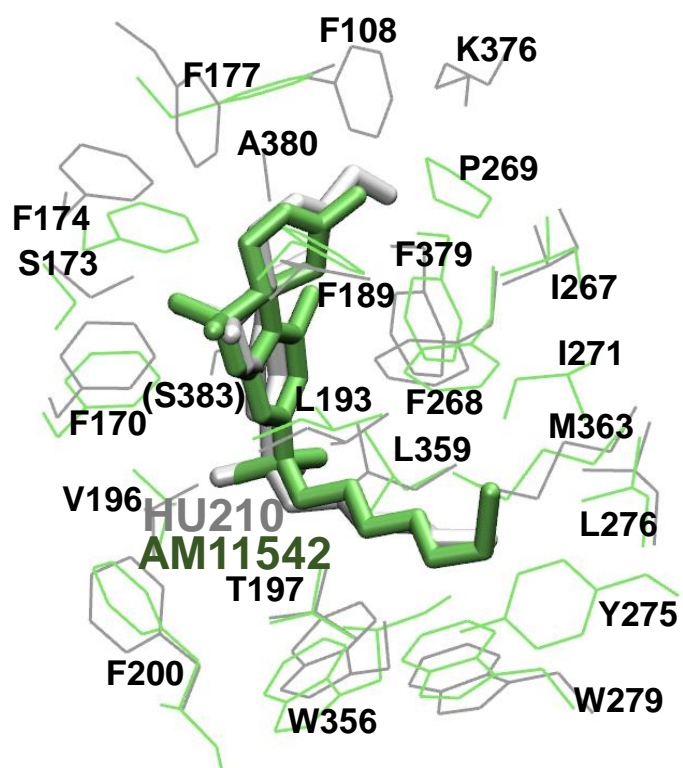

Supplement: S1 Fig — HU210 (in gray) in the binding pocket of the CB1-Gi complex model [23], refined according to the X-ray crystal structure of the AM11542-bound CB1 receptor [6], is overlaid to AM11542 (in green) in the X-ray crystal structure of the AM11542-bound CB1 receptor [6]. The binding pocket residues within 4 Å of the ligand are also displayed. (PDF) [file pone.0229879.s001.pdf]

A

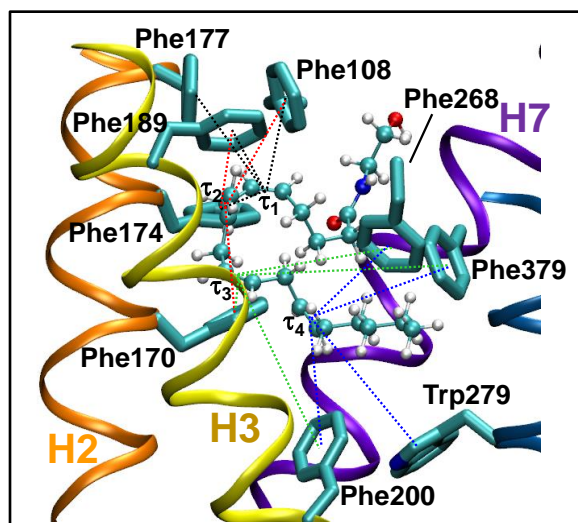

B

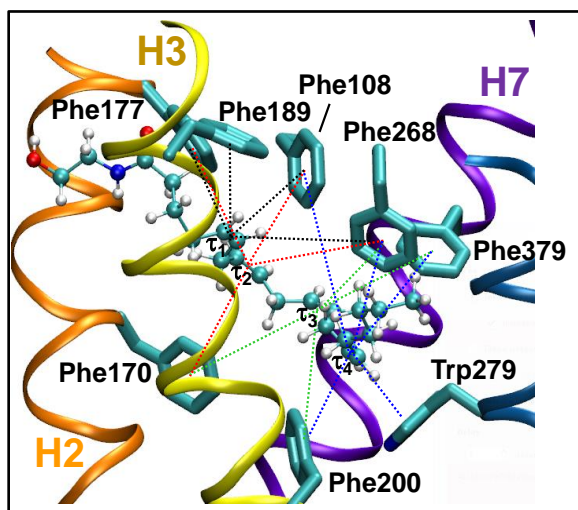

C

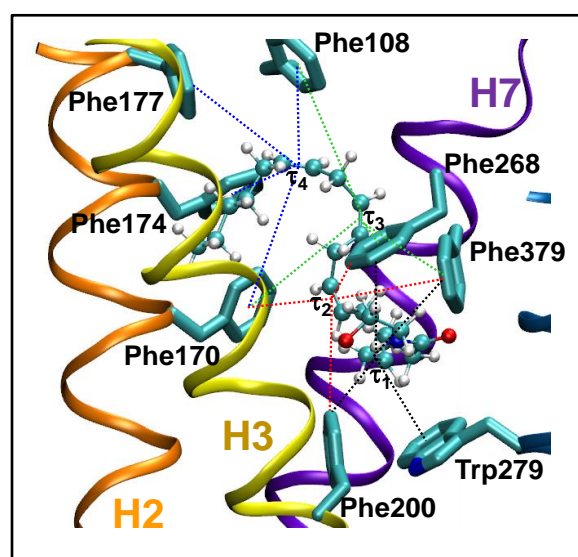

Supplement: S3 Fig — (A) AEA binding pose 1_H7_HC. (B) AEA binding pose 1_H2/H3_HC. (C) AEA binding pose 2_HCd_H2/H3. The aromatic-π stacking interactions are shown in the dotted lines. A criterion of 6 Å was used to approximate aromatic-π stacking interactions between the centroid of an aromatic ring and the centers of mass of the first double bond (τ1, C5 = C6) of AEA (in black), the second double bond (τ2, C8 = C9) of AEA (in red), the third double bond (τ3, C11 = C12) of AEA (in green) and the fourth double bond (τ4, C14 = C15) of AEA (in blue). (PDF) [file pone.0229879.s003.pdf]

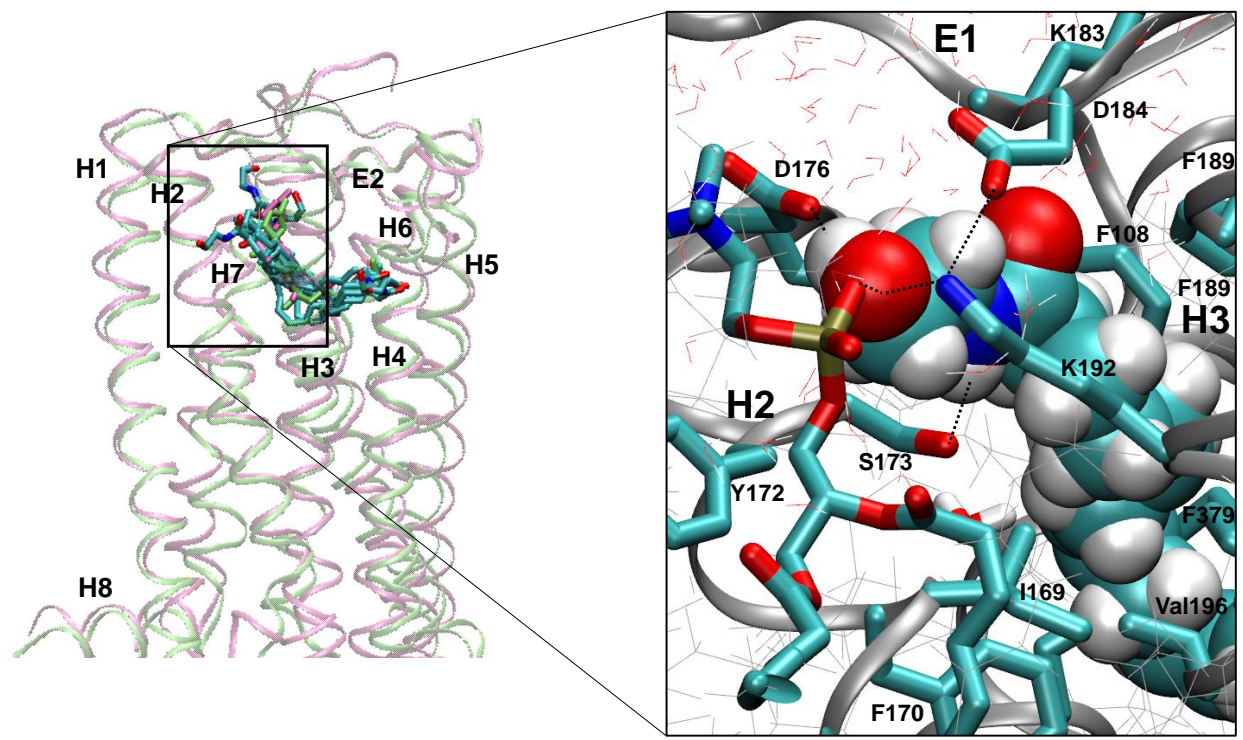

Supplement: S4 Fig — Hydrogen bonding interactions are shown in red dotted lines. Hydrogen bonding distance (in Å) is also shown. Residues and water molecules are shown in stick mode and AEA are shown in space-filling mode. (PDF) [file pone.0229879.s004.pdf]

**A**

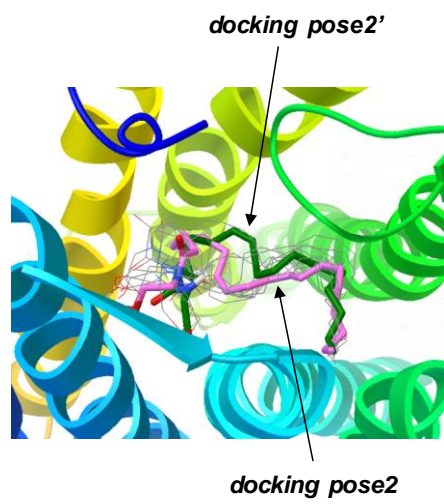

**B**

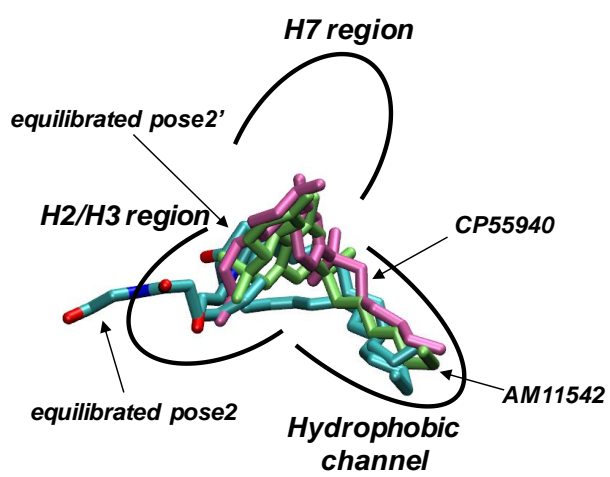

**C**

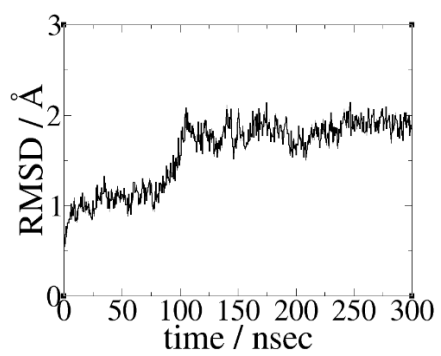

**D**

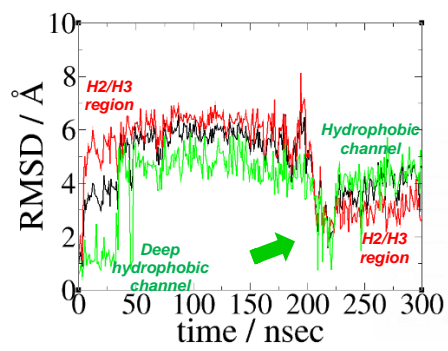

Supplement: S5 Fig — (A) Docking pose2’ (in dark green) and the AEA docking poses (in line mode) that belong to the same cluster as docking pose2 (in pink). (B) AEA binding pose 1_H2/H3_HC (equilibrated pose2 and equilibrated pose2’) overlaid to AM11542 (in green) and CP55940 (in mauve) in the X-ray crystal structures of the AM11542-bound CB1 receptor [6] and the CP55940-bound CB1 receptor [7]. (C) The RMSD values of the CB1 receptor in docking pose2’. (D) The RMSD plots of the head moiety and the tail moiety of AEA in docking pose2’. The RMSD values of the polar head moiety (in red) and the hydrophobic tail moiety (in green) of the bound AEA (in red). (PDF) [file pone.0229879.s005.pdf]
