# Supplementary material for: Prediction of essential binding domains for the endocannabinoid N-arachidonoylethanolamine (AEA) in the brain cannabinoid CB1 receptor
Source: PLoS One. 2021 Jun 28;16(6):e0229879. doi: 10.1371/journal.pone.0229879 (PMC8238219; doi:10.1371/journal.pone.0229879)

A

(a) *docking pose1*

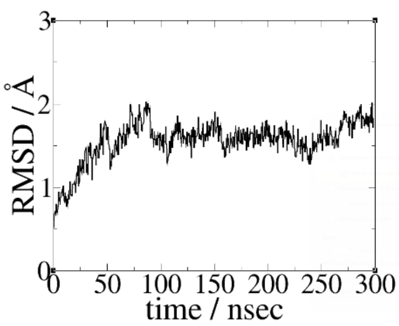

(b) *docking pose2*

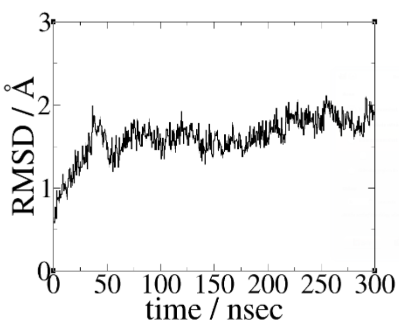

(c) *docking pose3*

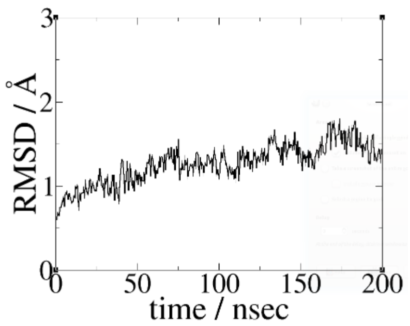

B

(a) *docking pose4*

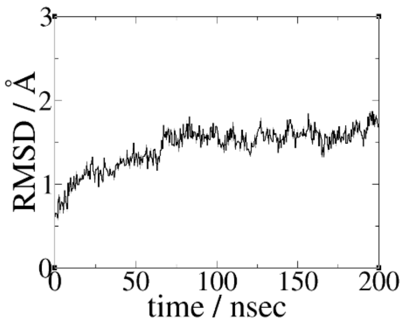

(b) *docking pose5*

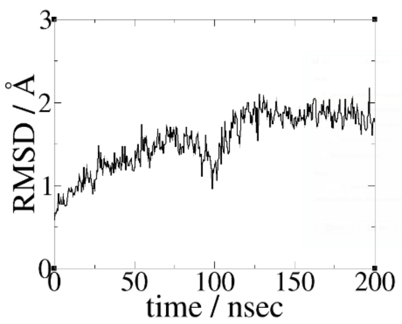

(c) *docking pose6*

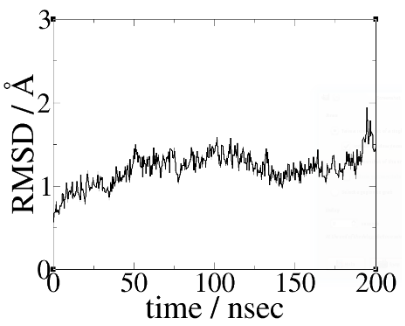

C

(a) *docking pose7*

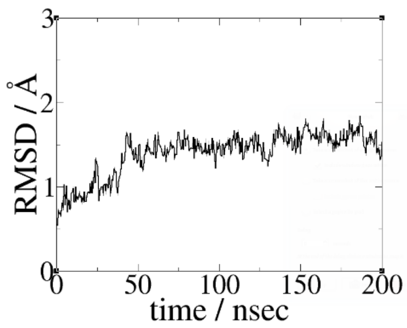

(b) *docking pose8*

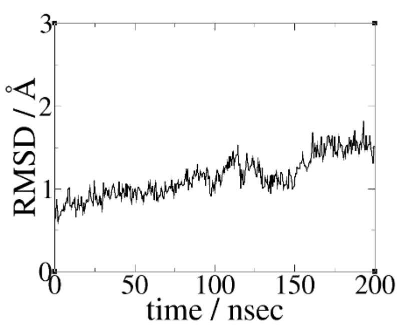

Supplement: S2 Fig — The RMSD values were calculated by root mean square fitting to the initial coordinates with respect to the backbone heavy atoms of the TM helical residues of the CB1 receptor. (A) AEA docking pose Group 1. (B) AEA docking pose Group 2. (C) AEA docking pose Group 3. (PDF) [file pone.0229879.s002.pdf]
