# Supplementary material for: Prediction of essential binding domains for the endocannabinoid N-arachidonoylethanolamine (AEA) in the brain cannabinoid CB1 receptor
Source: PLoS One. 2021 Jun 28;16(6):e0229879. doi: 10.1371/journal.pone.0229879 (PMC8238219; doi:10.1371/journal.pone.0229879)
Supplement: S1 Table — (PDF) [file pone.0229879.s006.pdf]

	Van der Waals interaction energies	Electrostatic interaction energies	Non-bonding interaction energies
AEA binding pose **1_H7_HC**			
Equilibrated *pose1*	-54.99(3.24)	-16.99(3.99)	-71.97(4.44)
Head[a)]	-18.38(2.19)	-13.41(3.86)	-31.79(3.42)
Polyene[b)]	-21.82(1.49)	-3.75(1.04)	-25.56(1.64)
Tail[c)]	-14.79(1.26)	0.17(0.36)	-14.62(1.19)
Equilibrated *pose3*	-52.42(3.77)	-29.46(7.44)	-81.88(6.46)
Head[a)]	-16.38(2.70)	-27.54(6.95)	-43.92(6.00)
Polyene[b)]	-21.98(1.54)	-2.14(1.15)	-24.12(1.71)
Tail[c)]	-14.06(1.18)	0.22(0.41)	-13.84(1.23)
Equilibrated *pose8*	-53.27(2.68)	-31.31(6.06)	-84.57(5.38)
Head[a)]	-16.13(2.45)	-29.31(5.99)	-45.45(4.62)
Polyene[b)]	-22.25(1.21)	-2.32(1.40)	-24.57(1.70)
Tail[c)]	-14.89(1.00)	0.33(0.34)	-14.56(1.05)
AEA binding pose **1_H2/H3_HC**			
Equilibrated *pose2*	-57.27(2.95)	-33.95(8.82)	-91.22(7.67)
Head[a)]	-19.01(2.57)	-30.87(8.32)	-49.38(6.99)
Polyene[b)]	-23.63(1.38)	-3.31(1.54)	-26.94(1.75)
Tail[c)]	-14.64(1.12)	0.23(0.32)	-14.40(1.14)
Equilibrated *pose2'*	-53.78(3.52)	-22.59(4/06)	-76.38(4.13)
Head[a)]	-17.75(2.64)	-21.24(4.01)	-38.99(3.68)

Polyene[b)]	-22.72(1.99)	-1.55(1.15)	-24.26(2.24)
Tail[c)]	-13.31(1.34)	0.19(0.32)	-13.12(1.38)
AEA binding pose **2_HC$_d$_H2/H3**			
Equilibrated *pose4*	-52.66(2.87)	-22.71(3.90)	-75.37(3.19)
Head[a)]	-17.26(1.89)	-20.23(4.02)	-37.50(3.08)
Polyene[b)]	-22.36(1.73)	-2.19(1.28)	-24.55(2.06)
Tail[c)]	-13.04(1.33)	-0.27(0.40)	-13.32(1.42)
Equilibrated *pose5*	-58.69(2.97)	-18.32(4.66)	-77.01(4.32)
Head[a)]	-20.59(2.06)	-15.20(4.44)	-35.79(3.73)
Polyene[b)]	-23.54(1.60)	-2.90(1.04)	-26.44(1.98)
Tail[c)]	-14.55(1.11)	-0.22(0.35)	-14.77(1.08)
Equilibrated *pose6*	-54.75(3.07)	-17.84(3.91)	-72.59(3.78)
Head[a)]	-19.47(2.45)	-15.20(3.74)	-34.67(3.08)
Polyene[b)]	-22.17(1.39)	-2.47(1.43)	-24.63(2.08)
Tail[c)]	-13.11(1.36)	-0.18(0.40)	-13.28(1.46)
Equilibrated *pose7*	-57.81(3.50)	-20.19(5.01)	-78.00(4.81)
Head[a)]	-20.30(2.19)	-17.57(5.13)	-37.87(4.18)
Polyene[b)]	-23.30(1.75)	-2.31(1.32)	-25.61(2.34)
Tail[c)]	-14.21(1.35)	-0.31(0.42)	-14.52(1.46)

[a]For the head moiety of the ligand, both the ethanolamide and the propyl (C2-C4) linker atoms were used.
[b]For the polyene moiety of the ligand, only the polyene (C5-C15) linker atoms were used.
[c]For the tail moiety of AEA, the pentyl (C16-C20) tail atoms were used.
