# Supplementary material for: Prediction of essential binding domains for the endocannabinoid N-arachidonoylethanolamine (AEA) in the brain cannabinoid CB1 receptor
Source: PLoS One. 2021 Jun 28;16(6):e0229879. doi: 10.1371/journal.pone.0229879 (PMC8238219; doi:10.1371/journal.pone.0229879)
Supplement: S2 Table — (PDF) [file pone.0229879.s007.pdf]

	Energy of the complex	Energy of the binding pocket residues	Energy of the ligand	Nonbonding interaction energy
AEA binding pose **1_H7_HC**				
Equilibrated *pose1*(+1)[a)]	-1757.21	-1658.84	-69.02	-29.35
Equilibrated *pose3*(+1)[a)]	-1624.84	-1522.59	-68.27	-33.98
Equilibrated *pose8*(0)[a)]	-2103.49	-2005.02	-69.20	-29.27
AEA binding pose **1_H2/H3_HC**				
Equilibrated *pose2*(0)[a)]	-2684.86	-2581.97	-66.14	-36.75
Equilibrated *pose2'*(0)[a)]	2141.77	-2039.22	-66.16	-36.39
AEA binding pose **2_HC$_{\mathbf{d}}$_H2/H3**				
Equilibrated *pose4*(+1)[a)]	-1594.56	-1500.03	-69.91	-24.62
Equilibrated *pose5*(0)[a)]	-2010.28	-1912.38	-69.06	-28.84
Equilibrated *pose6*(+1)[a)]	-1797.92	-1705.93	-66.90	-25.09
Equilibrated *pose7*(+1)[a)]	-1756.56	-1658.92	-69.24	-28.40

[a]The value in the parenthesis shows the total charge of the system.
