# Supplementary material for: Prediction of essential binding domains for the endocannabinoid N-arachidonoylethanolamine (AEA) in the brain cannabinoid CB1 receptor
Source: PLoS One. 2021 Jun 28;16(6):e0229879. doi: 10.1371/journal.pone.0229879 (PMC8238219; doi:10.1371/journal.pone.0229879)
Supplement: S3 Table — (PDF) [file pone.0229879.s008.pdf]

	Energy of the complex	Energy of the binding pocket residues	Energy of the ligand	Nonbonding interaction energy
AEA binding pose **1_H7_HC**				
Equilibrated *pose1*(+1)[a)]	-9234134.25	-8565637.49	-668429.97	-66.79
Equilibrated *pose3*(+1)[a)]	-10272217.71	-9603710.33	-668425.73	-81.65
Equilibrated *pose8*(0)[a)]	-9033473.83	-8364971.76	-668429.14	-72.93
AEA binding pose **1_H2/H3_HC**				
Equilibrated *pose2*(0)[a),b)]	-10604884.22	-9936366.58	-668421.69	-95.95
Equilibrated *pose2'*(0)[a)]	-10313509.70	-9645002.27	-668430.73	-76.70
AEA binding pose **2_HC$_d$_H2/H3**				
Equilibrated *pose4*(+1)[a)]	-9195744.65	-8527251.83	-668423.95	-68.87
Equilibrated *pose5*(0)[a)]	-10114011.17	-9445508.78	-668424.04	-78.36
Equilibrated *pose6*(+1)[a)]	-10044465.77	-9375972.62	-668422.09	-71.06
Equilibrated *pose7*(+1)[a)]	-9863215.22	-9194715.78	-668425.15	-74.29

[a]The value in the parenthesis shows the total charge of the system.
[b]The lipid tail, which was not involved in ligand interactions, was replaced by the methyl group.
