# Supplementary material for: Prediction of essential binding domains for the endocannabinoid N-arachidonoylethanolamine (AEA) in the brain cannabinoid CB1 receptor
Source: PLoS One. 2021 Jun 28;16(6):e0229879. doi: 10.1371/journal.pone.0229879 (PMC8238219; doi:10.1371/journal.pone.0229879)
Supplement: S1 File — (PDF) [file pone.0229879.s009.pdf]

! CHARMM parameters for anandamide (AEA)

```
BOND
NH1  CTL2       320.000     1.4300
NH1  CL         370.000     1.3450

ANGLE
H    NH1  CL      34.000   123.0000
H    NH1  CTL2    35.000   117.0000
CTL2 NH1  CL      50.000   120.0000
NH1  CTL2 CTL2    70.000   113.5000
NH1  CTL2 HAL2    51.500   109.5000
OBL  CL   NH1     80.000   122.5000
NH1  CL   CTL2    80.000   116.5000

DIHEDRAL
HAL2   CTL2  NH1  H        0.0000  3     0.00
OBL    CL    NH1  CTL2     2.5000  2   180.00
OBL    CL    NH1  H        2.5000  2   180.00
H      NH1   CTL2 CTL2     0.0000  1     0.00
CTL2   CTL2  NH1  CL       1.8000  1     0.00
CTL2   CL    NH1  CTL2     1.6000  1     0.00
CTL2   CL    NH1  CTL2     2.5000  2   180.00
HAL2   CTL2  NH1  CL       0.0000  3     0.00
H      NH1   CL   CTL2     2.5000  2   180.00

IMPROPER
OBL    X    X    CL     120.0000  0     0.0000
NH1    X    X    H       20.0000  0     0.0000
NH1    X    X    CTL2    20.0000  0     0.0000
```
