# Supplementary material for: Prediction of essential binding domains for the endocannabinoid N-arachidonoylethanolamine (AEA) in the brain cannabinoid CB1 receptor
Source: PLoS One. 2021 Jun 28;16(6):e0229879. doi: 10.1371/journal.pone.0229879 (PMC8238219; doi:10.1371/journal.pone.0229879)
Supplement: S2 File — (PDF) [file pone.0229879.s010.pdf]

! Topology definitions for anandamide (AEA)

```
RESI AEA          0.00 !
GROUP                  !
ATOM C5   CTL2    0.05 !
ATOM H51  HAL2    0.09 !                  OH1---HO1
ATOM H52  HAL2    0.09 !                  |
ATOM OH1  OHL    -0.66 !                  |
ATOM HO1  HOL     0.43 !            H52---C5---H51
GROUP                  !                  |
ATOM N1   NH1    -0.47 !                  |
ATOM HN1  H       0.31 !            H12---C1---H11
ATOM C1   CTL2   -0.22 !                  |
ATOM H11  HAL2    0.09 !                  |
ATOM H12  HAL2    0.09 !                  N1---HN1
ATOM C21  CL      0.79 !                 /
ATOM O22  OBL    -0.55 !                /
ATOM C22  CTL2   -0.22 !         O22==C21
ATOM H2R  HAL2    0.09 !               |
ATOM H2S  HAL2    0.09 !               |
GROUP                  !         H2R---C22---H2S
ATOM C23  CTL2   -0.18 !               |
ATOM H3R  HAL2    0.09 !               |
ATOM H3S  HAL2    0.09 !         H3R---C23---H3S
GROUP                  !               |
ATOM C24  CTL2   -0.18 !               |
ATOM H4R  HAL2    0.09 !         H4R---C24---H4S
ATOM H4S  HAL2    0.09 !               |
GROUP                  !               |
ATOM C25  CEL1   -0.15 !               |
ATOM H5R  HEL1    0.15 !         H5R---C25
GROUP                  !               ||
ATOM C26  CEL1   -0.15 !               ||
ATOM H6R  HEL1    0.15 !         H6R---C26
GROUP                  !               |
ATOM C27  CTL2   -0.18 !               |
ATOM H7R  HAL2    0.09 !         H7R---C27--H7S
ATOM H7S  HAL2    0.09 !               |
GROUP                  !               |
ATOM C28  CEL1   -0.15 !               |
ATOM H8R  HEL1    0.15 !         H8R---C28
GROUP                  !               ||
ATOM C29  CEL1   -0.15 !               ||
ATOM H9R  HEL1    0.15 !         H9R---C29
GROUP                  !               |
ATOM C210 CTL2   -0.18 !               |
ATOM H10R HAL2    0.09 !               |
ATOM H10S HAL2    0.09 !       H10R---C210--H10S
GROUP                  !              |
ATOM C211 CEL1   -0.15 !              |
```

```
ATOM H11R HEL1     0.15 !       H11R---C211
GROUP                   !              ||
ATOM C212 CEL1   -0.15 !              ||
ATOM H12R HEL1     0.15 !       H12R---C212
GROUP                   !              |
ATOM C213 CTL2   -0.18 !              |
ATOM H13R HAL2     0.09 !       H13R---C213--H13S
ATOM H13S HAL2     0.09 !              |
GROUP                   !              |
ATOM C214 CEL1   -0.15 !              |
ATOM H14R HEL1     0.15 !       H14R---C214
GROUP                   !              ||
ATOM C215 CEL1   -0.15 !              ||
ATOM H15R HEL1     0.15 !       H15R---C215
GROUP                   !              |
ATOM C216 CTL2   -0.18 !              |
ATOM H16R HAL2     0.09 !       H16R---C216--H16S
ATOM H16S HAL2     0.09 !              |
GROUP                   !              |
ATOM C217 CTL2   -0.18 !              |
ATOM H17R HAL2     0.09 !       H17R---C217--H17S
ATOM H17S HAL2     0.09 !              |
GROUP                   !              |
ATOM C218 CTL2   -0.18 !              |
ATOM H18R HAL2     0.09 !       H18R---C218--H18S
ATOM H18S HAL2     0.09 !              |
GROUP                   !              |
ATOM C219 CTL2   -0.18 !              |
ATOM H19R HAL2     0.09 !       H19R---C219--H19S
ATOM H19S HAL2     0.09 !              |
GROUP                   !              |
ATOM C220 CTL3   -0.27 !              |
ATOM H20R HAL3     0.09 !       H20R---C220--H20S
ATOM H20S HAL3     0.09 !              |
ATOM H20T HAL3     0.09 !             H20T
BOND C1 H11 C1 H12 C1 C5 C1 N1
BOND C5 H51 C5 H52
BOND C5 OH1 OH1 HO1
BOND N1 HN1 N1 C21
DOUBLE C21  O22
BOND   C21  C22
BOND   C22  C23    C22  H2R     C22  H2S
BOND   C23  C24    C23  H3R     C23  H3S
BOND   C24  C25    C24  H4R     C24  H4S
DOUBLE C25  C26
BOND   C25  H5R
BOND   C26  C27    C26  H6R
BOND   C27  C28    C27  H7R     C27  H7S
DOUBLE C28  C29
BOND   C28  H8R
BOND   C29  C210   C29  H9R
BOND   C210 C211   C210 H10R    C210 H10S
```

```
DOUBLE C211 C212
BOND   C211 H11R
BOND   C212 C213   C212 H12R
BOND   C213 C214   C213 H13R    C213 H13S
DOUBLE C214 C215
BOND   C214 H14R
BOND   C215 C216   C215 H15R
BOND   C216 C217   C216 H16R    C216 H16S
BOND   C217 C218   C217 H17R    C217 H17S
BOND   C218 C219   C218 H18R    C218 H18S
BOND   C219 C220   C219 H19R    C219 H19S
BOND   C220 H20T   C220 H20R    C220 H20S
IMPR C21  N1   C22   O22   C21  C22   N1  O22
IMPR N1   C21  HN1   C1    N1   C21   C1  HN1
IC N1    C1    C5    OH1    1.5084  107.86   48.44  104.92   1.3987
IC C1    C5    OH1   HO1    1.5191  104.92  173.19  111.99   0.9495
IC HN1   N1    C1    C5     1.0145  108.35 -148.00  107.86   1.5191
IC N1    C5    *C1   H11    0.0000    0.00  120.00    0.00   0.0000
IC N1    C5    *C1   H12    0.0000    0.00 -120.00    0.00   0.0000
IC OH1   C1    *C5   H51    0.0000    0.00  120.00    0.00   0.0000
IC OH1   C1    *C5   H52    0.0000    0.00 -120.00    0.00   0.0000
IC C1    N1    C21   C22    1.2588  122.06 -147.80  117.66   1.5191
IC C1    N1    C21   O22    1.2588  122.06 -147.80   32.52   1.2506
IC O22   C22   C21*  N1     0.0000    0.00  180.00    0.00   0.0000
IC HN1   N1    C21   O22    1.0145  122.10  180.00  122.10   1.2506
IC C23   C21   *C22  H2R    1.5483  113.78 -121.17  107.02   1.1096
IC C23   C21   *C22  H2S    1.5483  113.78  122.25  107.54   1.1088
IC C21   C22   C23   C24    1.5329  113.78  180.00  112.27   1.5435
IC C24   C22   *C23  H3R    1.5435  112.27 -122.24  109.63   1.1133
IC C24   C22   *C23  H3S    1.5435  112.27  120.06  108.89   1.1154
IC C22   C23   C24   C25    1.5483  112.27  180.00  115.67   1.5107
IC C25   C23   *C24  H4R    1.5107  115.67 -121.06  107.11   1.1144
IC C25   C23   *C24  H4S    1.5107  115.67  124.06  108.43   1.1128
IC C23   C24   C25   C26    1.5435  115.67  180.00  125.97   1.3453
IC C26   C24   *C25  H5R    1.3453  125.97 -176.85  115.39   1.1011
IC C24   C25   C26   C27    1.5107  125.97    0.00  125.28   1.5097
IC C27   C25   *C26  H6R    1.5097  125.28  178.19  119.65   1.1004
IC C25   C26   C27   C28    1.3453  125.28  180.00  121.35   1.5192
IC C28   C26   *C27  H7R    1.5192  121.35 -124.15  108.68   1.1135
IC C28   C26   *C27  H7S    1.5192  121.35  123.34  106.97   1.1121
IC C26   C27   C28   C29    1.5097  121.35  180.00  132.80   1.3549
IC C29   C27   *C28  H8R    1.3549  132.80 -178.43  111.35   1.1010
IC C27   C28   C29   C210   1.5192  132.80    0.00  130.38   1.5115
IC C210  C28   *C29  H9R    1.5115  130.38  178.53  117.07   1.1014
IC C28   C29   C210  C211   1.3549  130.38  180.00  111.80   1.5083
IC C211  C29   *C210 H10R   1.5192  121.35 -124.15  108.68   1.1135
IC C211  C29   *C210 H10S   1.5192  121.35  123.34  106.97   1.1128
IC C29   C210  C211  C212   1.5115  111.80  180.00  124.32   1.3436
IC C212  C210  *C211 H11R   1.3453  125.97 -176.85  115.39   1.1011
IC C210  C211  C212  C213   1.5083  124.32    0.00  125.45   1.5067
IC C213  C211  *C212 H12R   1.5097  125.28  178.19  119.65   1.1004
IC C211  C212  C213  C214   1.3436  125.45  180.00  111.57   1.5090
```

```
IC C214  C212  *C213 H13R   1.5192  121.35 -124.15  108.68   1.1135
IC C214  C212  *C213 H13S   1.5192  121.35  123.34  106.97   1.1128
IC C212  C213  C214  C215   1.5067  111.57  180.00  126.10   1.3471
IC C215  C213  *C214 H14R   1.3453  125.97 -176.85  115.39   1.1011
IC C213  C214  C215  C216   1.5090  126.10    0.00  125.86   1.5091
IC C216  C214  *C215 H15R   1.5097  125.28  178.19  119.65   1.1004
IC C214  C215  C216  C217   1.3471  125.86  180.00  113.25   1.5428
IC C217  C215  *C216 H16R   1.5192  121.35 -124.15  108.68   1.1135
IC C217  C215  *C216 H16S   1.5192  121.35  123.34  106.97   1.1128
IC C215  C216  C217  C218   1.5091  113.25  180.00  115.19   1.5395
IC C218  C216  *C217 H17R   1.5192  121.35 -124.15  108.68   1.1135
IC C218  C216  *C217 H17S   1.5192  121.35  123.34  106.97   1.1128
IC C216  C217  C218  C219   1.5428  115.19  180.00  113.95   1.5345
IC C219  C217  *C218 H18R   1.5192  121.35 -124.15  108.68   1.1135
IC C219  C217  *C218 H18S   1.5192  121.35  123.34  106.97   1.1128
IC C217  C218  C219  C220   1.5395  113.95  180.00  112.95   1.5309
IC C220  C218  *C219 H19R   1.5192  121.35 -124.15  108.68   1.1135
IC C220  C218  *C219 H19S   1.5192  121.35  123.34  106.97   1.1128
IC C218  C219  C220  H20T   1.5345  112.95  180.00  110.39   1.1115
IC H20T  C219  *C220 H20R   1.5192  121.35 -124.15  108.68   1.1135
IC H20T  C219  *C220 H20S   1.5192  121.35  123.34  106.97   1.1128

PATCHING FIRST NONE LAST NONE
```
